# Development and Validation of a Method for Vitamin B_12_ Measurement in Nutritional Products by HPLC-ICP-MS

**DOI:** 10.1007/s12161-025-02882-z

**Published:** 2025-08-20

**Authors:** Mesay M. Wolle, Jordan Escavage, Patrick J. Gray

**Affiliations:** 1 Office of Chemistry and Toxicology, Office of Laboratory Operations and Applied Science, Human Foods Program, U.S. Food and Drug Administration, 5001 Campus Drive, College Park, MD 20740 USA; 2 Joint Institute for Food Safety and Applied Nutrition (JIFSAN), University of Maryland, College Park, MD 20742 USA

**Keywords:** Vitamin B_12_, Nutritional products, HPLC-ICP-MS, Validation

## Abstract

A method has been developed and validated for vitamin B_12_ (cobalamin) measurement in nutritional products (infant formula, toddler and adult nutritive drinks, milk, protein drinks/powders, and breakfast cereals). The optimum procedure was based on collective extraction of the various forms of vitamin B_12_ as cyanocobalamin (CNCbl) with a sodium acetate buffer (0.25 mol L^−1^, pH 4.5) at 95 °C for 30 min in the presence of sodium cyanide, followed by analyte concentration and cleanup on a solid phase extraction (SPE) sorbent. Starch-containing samples were treated with α-amylase (300 µL) and incubated (40 °C, 30 min) prior to heating at 95 °C. The SPE eluate was reconstituted to a smaller volume and subsequently analyzed using high-performance liquid chromatography coupled with inductively coupled plasma mass spectrometry (HPLC-ICP-MS). The analyte was separated isocratically on a C-8 column in under 6 minutes using a mobile phase of 0.1% (*v/v*) formic acid in methanol and water (30 : 70). The method was single laboratory validated according to U.S. Food and Drug Administration’s (FDA) guidelines and compared favorably to AOAC Official Methods 952.20 and 2014.02. The repeatability of the method (< 14% RSD) was demonstrated by interday analysis of samples and reference materials. Recovery values mostly ranged between 80% and 120% for fortified samples and reference materials. Vitamin B_12_ can be detected and quantified using the current method at levels as low as 0.004 and 0.03 µg CNCbl/100 g (liquid and ready-to-feed/drink samples) and 0.01 and 0.06 µg CNCbl/100 g (solid samples), respectively. The method is simpler and less time-consuming than most official and other published methods with broader application and better analytical performance.

## Introduction

Vitamin B_12_, also known as cobalamin, is an essential micronutrient which plays a key role in red blood cell formation and functioning of the brain and nervous system (Allen 2012). Its deficiency is manifested by pernicious anemia (Stabler and Allen 2004) and has been associated with neurological degeneration in infants (Dror and Allen 2008). Vitamin B_12_ refers to several molecular variants among which methylcobalamin (MeCbl) and 5-deoxyadenosylcobalamin (AdoCbl) are active in human metabolism. The other two forms, hydroxycobalamin (OHCbl) and cyanocobalamin (CNCbl), become biologically active when transformed to the coenzyme forms (NIH 2024). The synthetic and chemically stable form of cobalamin, CNCbl, is primarily used for food fortification and supplementation (Jägerstad and Arkbåge 2003). Humans cannot synthesize vitamin B_12_ in vivo and rely on dietary intake. Naturally occurring cobalamins are only found in animal products such as meat, liver, eggs, dairy, and fish (Rakuša et al. 2022) but its intake can be increased by consumption of fortified nutritional products. The recommended vitamin B_12_ daily intake varies with age and gender, from 0.4 μg in infants to 2.4 μg in adults, and those requirements increase up to 2.8 μg for pregnant and lactating women (NIH 2024).

The measurement of vitamin B_12_ in nutritional products has been mostly relying on methods applying microbiological assays, radioisotopic dilution, immunoassay, spectrophotometry, and liquid chromatography coupled with ultraviolet detection (LC-UV), many of which are limited in sensitivity and specificity, and/or laborious and time-consuming (Li et al. 2019; Tsiminis et al. 2017). In addition, most official (AOAC 1960, 2014) and other methods (Amritkar et al. 2020; Dubascoux et al. 2021; Pérez-Fernández et al. 2016; Yang et al. 2024) address only a limited number and types of products with a focus on infant formula and milk. The determination of vitamin B_12_ in nutritional products represents a challenging analytical task because these matrices are very complex and disparate, and the nutrient is often a microconstituent existing in multiple vitamers of wide-ranging stabilities (Li et al. 2019). Demand for rapid, specific, and updated methodologies is growing.

The unique structure of vitamin B_12_ containing a corrin system with a central cobalt (Co) provides an opportunity for selective detection using techniques normally dedicated to elemental analysis. Inductively coupled plasma-mass spectrometry (ICP-MS) is best suited for such analyses, providing sensitive, selective, and robust detection. Coupling high-performance liquid chromatography (HPLC) to ICP-MS to determine Co as a proxy for vitamin B_12_ quantification provides extremely selective detection as the measurement is accomplished by monitoring a single atom. The use of HPLC-ICP-MS has been used to determine vitamin B_12_ mainly in dietary supplements (Raju et al. 2013; Yang et al. 2014; Zhang et al. 2008) and biological matrices (Bosle et al. 2016; Czerwonka et al. 2014; Dubascoux et al. 2021; Honda et al. 2002; Szterk et al. 2012; Wenzel et al. 2018) with infrequent application in nutritional products (Raju et al. 2013; Yang et al. 2024).

The present study aimed to develop an HPLC-ICP-MS method for vitamin B_12_ measurement in infant formula, toddler and adult nutritive drinks, milk, protein drink/powder, and breakfast cereals. Sample preparation was based on liquid extraction followed by cleanup and analyte enrichment on a solid-phase extraction (SPE) sorbent. The method was single laboratory validated following U.S. Food and Drug Administration’s (FDA) guidelines (FDA 2019) and compared with AOAC Official Methods 952.20 (microbiological assay) (AOAC 1960) and 2014.02 (LC-UV) (AOAC 2014) on samples and reference materials covering a wide range of matrices and analyte concentrations.

## Experimental

### Reagents and Standards

Sodium acetate (≥ 99%), formic acid (98–100%), pepsin from porcine gastric mucosa, and α-amylase from *Aspergillus oryzae* (≥ 800 FAU/g) were purchased from MilliporeSigma. Optima grade nitric acid (HNO_3_, 67–70%), glacial acetic acid (99%)**,** hydrogen peroxide solution (H_2_O_2_, 30–32%), and sodium cyanide (98%) were purchased from Fisher Scientific. HPLC grade methanol and acetonitrile, and electronic grade 2-propanol were obtained from J.T. Baker Chemicals, and trifluoroacetic acid (≥ 99.5%) from Alfa Aesar. Water deionized to > 18 MΩ·cm (Milli-Q Element, Millipore) was used throughout. The absence of vitamin B_12_ in pepsin and α-amylase was confirmed through analysis by HPLC-ICP-MS.

**Fig. 1:**
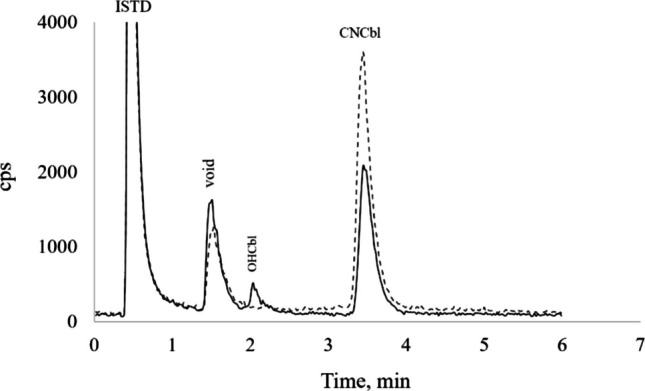
Chromatograms showing separation of CNCbl in a standard solution of 2.3 µg CNCbl/100 g (1.0 ng g^−1^ Co) (solid line) and in extract generated from an infant formula sample (dashed line). ISTD: post-column marker

USP grade AdoCbl, CNCbl, OHCbl**,** and MeCbl were purchased from US Pharmacopoeia, and a 10,025 μg mL^−1^ stock solution of Co from Inorganic Ventures. Stock standard solutions (100 µg g^−1^ in Co) of the four cobalamins were separately prepared by dissolving the required amounts of the solids to 25 g in water. The solutions were tightly sealed and stored in amber bottles at 4 °C. The CNCbl stock standard was analyzed by ICP-MS to verify the total Co mass fraction and was evaluated biweekly for degradation by monitoring the formation of OHCbl (Fig. 1); the solution was discarded if the OHCbl peak was > 2% relative to the CNCbl peak. Due to their lack of stability, the solutions of the other cobalamins were prepared when needed and used only for qualitative analysis. Since vitamin B_12_ is sensitive to light, standard solutions and extracts were prepared and stored under subdued light or in amber tubes.

### Samples and Reference Materials

A convenience sampling of 38 products, including seven infant formulas, ten toddler and adult nutritive drinks, six milks, eight protein powders/drinks, and seven breakfast cereals, was purchased from local stores (Table 1).

**Table 1:** List of samples with their label claim and measured vitamin B_12_ mass fractions

**Nutritional product**	**Sample ID**	**Matrix**	**Sample form**	**Vitamin B**_**12**_**, µg/100 g sample**		
				**Label claim**^**a**^	**Measured**	**%RSD**^**e**^
Infant formula	IF-MLK	Milk	Powder	1.3 (0.3)^b^	4.3	3.5
	IF-RTF	Milk	RTF	0.15 (0.25)^b^	0.6	3.1
	IF-SOY	Soy	Powder	2.1 (0.47)^b^	4.7	6.9
	IF-PHM	Partially hydrolyzed milk	Powder	1.4 (0.3)^b^	3.3	5.7
	IF-PHS	Partially hydrolyzed soy	Powder	1.3 (0.3)^b^	2.2	3.4
	IF-ST	Starch (potato)	Powder	1.3 (0.3)^b^	3.2	3.7
	IF-AA	Amino acid based, starch	Powder	1.2 (0.27)^b^	2.4	6.3
Toddler nutritive drink	TF-MLK-1	Whole milk	Powder	1.9 (0.5)^c^	5.0	5.9
	TF-MLK-2	Nonfat milk	Powder	1.1 (0.3)^c^	2.0	7.4
	TF-PLT	Plant-based, starch	Powder	2.3 (0.36)^c^	3.9	1.6
	TF-AA	Amino acid based, starch	Powder	1.9 (1.9)^c^	1.9	11.6
Adult nutritive drink	AF-RTD-1	Regular protein	RTD	0.25 (25)^d^	1.5	6.6
	AF-RTD-2	High protein	RTD	0.23 (25)^d^	1.6	5.9
	AF-RTD-3	Maximum protein	RTD	0.04 (6)^d^	0.8	6.5
	AF-RTD-4	Fat free	RTD	0.32 (40)^d^	2.0	6.1
	AF-RTD-5	High protein, plant-based, dairy free	RTD	0.25 (25)^d^	1.0	13.3
	AF-PWD	High protein	Powder	0.70 (0.7)^c^	2.1	6.8
Milk	MLK-1	Reduced fat	Liquid	0.53 (1.3)^c^	0.3	1.8
	MLK-2	Whole milk	Liquid	0.50 (1.2)^c^	0.2	5.7
	MLK-3	Lactose free, reduced fat	Liquid	0.53 (1.3)^c^	0.3	2.0
	MLK-4	Oat	Liquid	0.49 (1.2)^c^	0.3	5.5
	MLK-5	Soy	Liquid	0.51 (1.2)^c^	0.8	6.9
	MLK-6	Coconut	Liquid	0.51 (1.2)^c^	1.1	0.7
Protein drink/powder	PD-RTD-1	Milk	RTD	0.14 (20)^d^	0.6	3.6
	PD-RTD-2	Milk, whey, casein	RTD	0.36 (50)^d^	0.5	0.4
	PD-RTD-3	Pea, rice	RTD	0.07 (10)^d^	ND	
	PD-RTD-4	Soy	RTD	0.11 (20)^d^	0.3±	7.9
	PD-PWD-1	Milk	Powder	0.52 (35)^d^	2.8	5.7
	PD-PWD-2	Whey	Powder	0.46 (1.56)^c^	2.2	12.5
	PD-PWD-3	Casein	Powder	3.8 (1.21)^c^	8.5	5.1
	PD-PWD-4	Pea, flax, cranberry, baobab, moringa, chia, pumpkin	Powder	521 (5210)^d^	786	1.8
Breakfast cereal	CER-C	Corn	Flakes	1.7 (30)^d^	2.7	8.3
	CER-R	Rice	Flakes	1.8 (30)^d^	2.9	4.3
	CER-W	Wheat	Flakes	1.3 (20)^d^	2.8	1.9
	CER-O	Oat	Flakes	2.3 (40)^d^	2.5	9.3
	CER-M-1	Corn, rice, oat	Flakes	4.0 (90)^d^	4.1	9.1
	CER-M-2	Wheat, barley	Flakes	5.6 (100)^d^	7.3	3.3
	CER-M-3	Oat, sorghum	Flakes	2.1 (35)^d^	22	7.8

RTF: ready-to-feed, RTD: ready-to-drink, ND: not detected

^a^Vitamin B_12_ mass fraction (µg/100 g sample) calculated from label mass fractions (in parenthesis) given in ^b^µg/100 calories, ^c^µg/serving size, and ^d^% daily value/serving size; ^e^%RSD for measured vitamin B_12_ mass fraction (*n* = 3)

Standard reference materials (SRM) of infant formula (1846), infant/adult nutritional formula (1869), whole milk powder (1549a), soy milk (3235), protein drink mix (3252), and breakfast cereal (3233) were purchased from the National Institute of Standards and Technology (NIST). Certified reference material (CRM) of whole milk powder (ERM-BD600) from the European Union and reference materials (RM) of infant (8260) and adult nutritional (8261) formula from NIST were also used. The reference materials were certified either for vitamin B_12_ or CNCbl. SRM 3233 is not certified for vitamin B_12_; however, a value reported in a study from NIST was used as reference (Raju et al. 2013).

### Sample Homogenization

Powder samples (25 g) were homogenized, and flakes (25 g) were ground to fine powder using IKA Tube Mill 100 with disposable grinding chambers and then preserved in amber centrifuge tubes. Samples were analyzed without moisture correction. Liquid and ready-to-feed/drink (RTF/D) samples were shaken before taking analytical portions to ensure homogeneity. All samples were stored per label instructions. Refrigerated samples were brought to room temperature before use.

### Total Cobalt Measurement

Total Co was determined according to the method in FDA’s Elemental Analysis Manual Section 4.7 (FDA 2020). Samples were acid-digested in a Multiwave 7000 microwave (Anton Paar) and analyzed by Agilent 8800 ICP-MS. Analytical solutions were introduced using an ASX-500 Series autosampler (Agilent Technologies) and mixed 1 : 1, in a Teflon Tee-fitting, with a Ge internal standard solution (20 ng g^−1^) prepared in 5% (*v/v*) HNO_3_, 0.5% (*v/v*) HCl**,** and 4% (*v/v*) isopropanol. See Table 2 for the ICP-MS operating conditions.

**Table 2:** ICP-MS and HPLC-ICP-MS operating conditions

**ICP-MS**^**a**^	
RF power	1550 W
RF matching	1.8^b^, 1.4^c^ V
Sampling depth	8 mm
Plasma gas	15 L min^−1^
Carrier gas	1.04^b^, 0.7^c^ L min^−1^
Makeup gas	0.15 L min^−1^
Option gas mix^d^	20%^c^
ORS^3^ gas (He) flow	4.5^b^, 2.0^c^ mL min^−1^
Plasma ignition mode	aqueous solution^b^, organic solvent^c^
Spray chamber temperature	2°C
Nebulizer	Concentric
Torch injector	2.5^b^, 1.5^c^ mm i.d. quartz
Sampler/skimmer cones	Ni^b^, Pt^c^
Data acquisition	Spectrum^b^, time resolved analysis (TRA)^c^
Integration time	0.5^b^, 1.0^c^ s
Peak pattern	3 points per mass^b^
Replicates per ion	9^b^, 1^c^
**HPLC**	
Analytical column^e^	Agilent Zorbax Eclipse XDB-C8 (3 x 150 mm, 3.5 μm)
Mobile phases	0.1% formic acid in (A) water and (B) methanol
Isocratic	0–6.0 min (30% B)
Flow rate	0.4 mL/min
Autosampler temperature	4 °C
Injection volume	20 μL
Column temperature	Ambient
Sample diluent	Mobile phase A

^a^Ultrahigh purity (99.999%) argon and oxygen–argon mix (20:80) from Roberts Oxygen Company and helium from Airgas were used

^b^Total Co by ICP-MS (Agilent 8800)

^c^Vitamin B_12_ by HPLC-ICP-MS (Agilent 7900)

^d^Oxygen in argon (20 : 80)

^e^Column was rinsed with 95% (*v/v*) methanol for 20–30 min at the start of every analytical batch; fresh column was conditioned for 3 h

### Microbiological Assay

Microbiological assays were performed by two external laboratories according to AOAC Official Method 952.20 (AOAC 1960).

### UPLC-UV Analysis

Analysis was carried out according to AOAC Official Method 2014.02 (AOAC 2014). A Waters Acquity ultraperformance liquid chromatographic (UPLC) system with Waters Acquity UPLC BEH C18 (2.1 × 100 mm, 1.7 µm) column and PDA eλ detector was used. Extracts were cleaned up and preconcentrated on EASI-EXTRACT Vitamin B_12_ LGE immunoaffinity column from R-Biopharm.

### Evaluation of Extraction Methods

Eight extraction methods described in Table 3 were evaluated. Depending on the level of vitamin B_12_, 0.5–2.0 g of reference material was mixed with 10 mL of extraction solution and vortexed for 30 seconds. For methods other than M4 and M5, starch-containing samples were treated with α-amylase (300 µL) and incubated in a shaking water bath (Boekel Scientific) at 40 °C for 30 minutes. Mixtures were then subjected to the corresponding extraction procedure in Table 3, cooled to room temperature**,** and centrifuged at 1960 x g for 15 minutes. Supernatants were filtered through a 0.45 µm Millex PVDF syringe filter (MilliporeSigma) and analyzed by HPLC-ICP-MS (Table 2). An ultrasonic cleaner (Branson) was used for sonication, and hot block extractions were performed using a DigiPREP MS 48-position temperature-controlled hot block (SCP Science).

**Table 3:** Extraction methods evaluated

Method	Extraction solution	Extraction, temp.	Time	Reference
M1	Water	Sonicate, ambient	15 min	(Raju et al. 2013)
M2	Sodium acetate, 0.25 mol L^−1^, pH 4.5^a^	Sonicate, ambient	15 min	(Pérez-Fernández et al. 2016)
M3	Phosphate buffer, 0.05 mol L^−1^, pH 5.8^b^	Sonicate, ambient	15 min	(Choi et al. 2003; Qiu et al. 2019)
M4	Acetic acid, 1% (*v/v*)	Sonicate, ambient	15 min	(D'Ulivo et al. 2017)
M5	Nitric acid, 1% (*v/v*)	Sonicate, ambient	15 min	(Chen and Jiang 2008)
M6	Pepsin in sodium acetate, 0.25 mol L^−1^, pH 4.5^a,c^	Water bath, 40 °C	180 min	(Heudi et al. 2006)
M7	Sodium acetate, 0.25 mol L^−1^, pH 4.5^a^	Hot block, 95 °C	30 min	
M8	Sodium acetate, 0.25 mol L^−1^, pH 4.5^a^ and sodium cyanide, 1% (*w/v*)^d^	Hot block, 95 °C	30 min	(Amritkar et al. 2020)

^a^pH adjusted with acetic acid

^b^Buffer was prepared from dipotassium hydrogen phosphate and potassium dihydrogen phosphate; pH adjusted with 1 mol L^−1^ sodium hydroxide solution

^c^500 mg pepsin in 10 mL acetate buffer

^d^250 µL of 1% (*w/v*) sodium cyanide in 10-mL acetate buffer

### Evaluation of Solid Phase Extraction Materials

Four SPE materials were compared for analyte enrichment and cleanup: EASI-EXTRACT Vitamin B_12_ LGE immunoaffinity column from R-Biopharm, Oasis HLB and Oasis PRiME HLB polymeric reversed phase sorbents (500 mg, 60 µm) from Waters Corporation, and Strata-X polymeric reversed phase sorbent (500 mg) from Phenomenex.

The storage buffer was drained from the immunoaffinity column, and the three reversed-phase materials were conditioned and equilibrated with methanol (3 mL) and water (3 mL), respectively. Then, a maximum of 9 mL (immunoaffinity column) or 15 mL (reversed phase columns) extract was loaded onto the sorbents. After all the extract was drained, the sorbents were rinsed with 3 mL water, and the analyte was eluted with 3 mL methanol into a centrifuge tube, followed by rinsing the sorbent with 0.5 mL methanol. Residual solvent on the sorbent was pushed into the tube by forcing air with a syringe. The SPE eluate was concentrated to dryness in a CentriVap benchtop vacuum concentrator (Labconco) at 55 °C and reconstituted in 0.5 mL of 0.1% (v/v) formic acid in water for subsequent analysis by HPLC-ICP-MS (Table 2). Solvents and extracts were loaded onto the sorbents manually and drained by gravity, except Oasis Prime HLB, which used a peristaltic pump for draining.

### Optimum Sample Preparation Procedure

Depending on the expected level of vitamin B_12_, a 0.5–2.0 g (solid) or 5.0 g (liquid and RTF/D) portion of sample was weighed into a 50-mL amber polypropylene centrifuge tube, and 10 mL of 0.25 mol L^−1^ sodium acetate (pH 4.5) was added. Starch-containing samples were treated with α-amylase (300 µL) and incubated in a water bath at 40 °C for 30 min under agitation. Then, 250 µL of 1% (*w/v*) sodium cyanide was added and the mixture was heated in a hot block for 30 min at 95 °C. After cooling to room temperature, the extraction mixture was centrifuged at 1960 x g for 15 min and the supernatant was filtered through a 0.45-µm syringe filter. The filtrate was subjected to SPE on an Oasis HLB sorbent and reconstituted as described in the previous section.

#### Analysis of Extracts

Analyses were performed using an Agilent 1260 HPLC coupled with an Agilent 7900 ICP-MS. Reversed-phase HPLC separation was carried out isocratically with a two-component mobile phase of 0.1% (*v/v*) formic acid in methanol and water (30:70). The ICP-MS was operated in O_2_/Ar option gas mode to oxidize the high amounts of carbon from methanol into CO_2_ and thereby avoid soot buildup on the cones. The collision cell was pressurized with helium to eliminate interference from polyatomic ions such as ^24^Mg^35^Cl, ^43^Ca^16^O, and ^23^Na^36^Ar on Co at *m/z* 59. A 5 ng g^−1^ Co solution, postcolumn injected using a switching valve, was used as internal standard (ISTD) to correct for instrument drift. The injection was timed so that the ISTD eluted before the void. Table 2 shows the HPLC-ICP-MS operating conditions.

#### Quantification

Quantifications were based on external calibrations, and concentrations were calculated and reported as mass fractions. For total Co measurement, standards (0.5–50 ng g^−1^ Co) were prepared by serially diluting the Co stock (10,025 µg mL^−1^) in 5% HNO_3_ and 0.5% HCl solution. For HPLC-ICP-MS analyses, calibration standards (0.5–20 ng g^−1^ Co) were prepared by serially diluting the CNCbl stock (100 µg g^−1^ Co) with 0.1% (*v/v*) formic acid in water. CNCbl standards were based on elemental Co mass fraction (as opposed to compound mass) and analyte mass fractions were finally reported as µg CNCbl/100 g. Standard solutions for UPLC-UV analyses were prepared from the CNCbl stock as described in AOAC Official Method 2014.02 (AOAC 2014). All calibration standards were prepared on the day of analysis**,** and dilutions were made gravimetrically.

Agilent MassHunter software was used for ICP-MS and HPLC-ICP-MS data acquisition, chromatographic peak integration**,** and analyte quantification. CNCbl was identified by matching the peak retention time with a reference standard**,** and its mass fractions were calculated based on peak area. Data were normalized to a Ge internal standard (ICP-MS) and post-column injected Co standard (HPLC-ICP-MS). Further calculations were performed using Microsoft Excel.

Waters Empower 3 Chromatography Data software was used for UPLC-UV data acquisition and chromatographic peak integration. Peak integrations were exported to Microsoft Excel for further processing.

#### Single Laboratory Validation

The limits of detection (LOD) and quantification (LOQ) of the method were determined according to the procedure in FDA’s Elemental Analysis Manual Section 3 (FDA 2021) based on 10 replicate injections of a fortified blank (deionized water)**.**

Extracts were generated from both the samples and the three-level fortified samples for each commercial product listed in Table 1 over the course of three days. The three-tiered spikes corresponded to 50% (low level), 100% (medium level), and 150% (high level) of the native analyte mass fraction in the sample (determined as CNCbl). For samples where vitamin B_12_ was not detected, the spiking amounts were established based on twice the limit of quantification (LOQ) of the method. Samples were fortified after weighing and before the addition of the extraction solution. Each extraction batch comprised a deionized water blank, two fortified blanks, samples, fortified samples, and matrix-matched reference material(s).

#### Safety

Cyanide is fatal if swallowed, inhaled, or comes in contact with skin. It reacts with acids to form highly toxic and rapid-acting hydrogen cyanide gas. An exhausting hood must be used with closed vessels when working with sodium cyanide.

## Results and Discussion

### Chromatographic Method

Some of the existing chromatographic methods for vitamin B_12_ are characterized by long analysis time (Chassaigne and Łobiński 1998; Heudi et al. 2006; Lee et al. 2015), or use salts such as EDTA (Raju et al. 2013; Yang et al. 2024) and phosphate (Qiu et al. 2019; Yang et al. 2024) which may lead to clogging of the HPLC system or gradual accumulation on the ICP-MS cones. Bosle et al. (2016) used a methanol–water gradient system to separate AdoCbl, CNCbl, OHCbl, and MeCbl on a C-8 column. This method was modified in the present study to separate CNCbl in a shorter time. Isocratic elution was preferred to ensure stable plasma conditions, and a reduced flow rate to avoid excessive carbon load. A 30 : 70 methanol–water mixture provided a good compromise in terms of analyte separation and run time. Formic acid (0.1% *v/v)* was added to ensure retention time reproducibility. Methanol concentration above 30% did not improve the shape of the CNCbl peak. The optimized condition (Table 2) separated CNCbl in less than 6 minutes (Fig. 1). Free Co was eluted within the void volume, while CNCbl was baseline separated from OHCbl; the latter serves as a reference to monitor the stability of CNCbl. The short analysis time enhances sample throughput. Since analyte detection is based on monitoring the Co in CNCbl (as opposed to molecular detection), a solution of Co was injected post-column to correct for instrument drift between sample injections. The fact that the analysis was performed in isocratic mode avoids the primary cause of signal drift, as it avoids change in mobile phase composition throughout the analysis.

### Extraction Method

As part of the identification of an optimum extraction condition for the samples of interest covering a wide range of matrices, the extraction methods described in Table 3 were evaluated by analyzing reference materials of infant/adult nutritional formula (SRM 1869), whole milk powder (ERM-BD600), protein drink mix (SRM 3252), and breakfast cereal (SRM 3233).

Fortified CNCbl can easily be solubilized through mild extraction using water (Raju et al. 2013) or buffer solutions (Choi et al. 2003), with the effectiveness of these methods assessed via sonication as described in Table 3 (M1–M3). Extraction of vitamin B_12_ has also been achieved through acidic precipitation of proteins (Oprean et al. 2011). In the present study, dilute solutions of acetic acid (M4) and nitric acid (M5) were evaluated via sonication. Proteolytic enzymes facilitate the release of cobalamins through biomolecular hydrolysis at moderate temperatures maintaining the native forms of analytes (Li et al. 2019). In M6, pepsin was used in acetate buffer, and the mixture was incubated at 40 °C in a shaking water bath for 3 h (Heudi et al. 2006).

Protein-bound cobalamins are also extracted by heating the sample in acetate buffer (Li et al. 2019). Due to the inherent instability of certain forms of cobalamin, a minimal quantity of cyanide is used to stabilize these analytes. Cyanidation is broadly acknowledged as an effective option for transforming cobalamins into a stable form (CNCbl) (Wang et al. 2023). Since it requires the application of heat, cyanidation is usually performed in conjunction with protein denaturation using acetate extraction. Cyanocobalamin exhibits maximum stability in the mild acidic region (Li et al. 2019), with cyanidation mostly performed at pH 4.0–4.5 in acetate buffer (Amritkar et al. 2020; AOAC 2014; Dubascoux et al. 2021; Rampazzo et al. 2022; Repossi et al. 2017; Wang et al. 2023; Zironi et al. 2013). In this study, acetate extraction was evaluated with (M8) and without cyanidation (M7).

Denaturation of proteins can also be achieved using acidified organic solvents (Reuter et al. 2016), but this procedure was not considered for evaluation as organic solvents interfere in analyte enrichment on SPE sorbent. In all the extraction procedures listed in Table 3, except for acidic extractions (M4 and M5), starch-containing samples were treated with α-amylase to enhance filtration by breaking down the matrix. All extracts from the methods in Table 3 were analyzed using the HPLC-ICP-MS method described in Table 2. Since SRMs 1869 and 3252 were certified for vitamin B_12_ instead of CNCbl specifically, their extracts were also analyzed using an HPLC-ICP-MS method that separates the four forms of cobalamin (unpublished) to account for other vitamin B_12_ variants than CNCbl that may be extracted from the matrices.

**Fig. 2:**
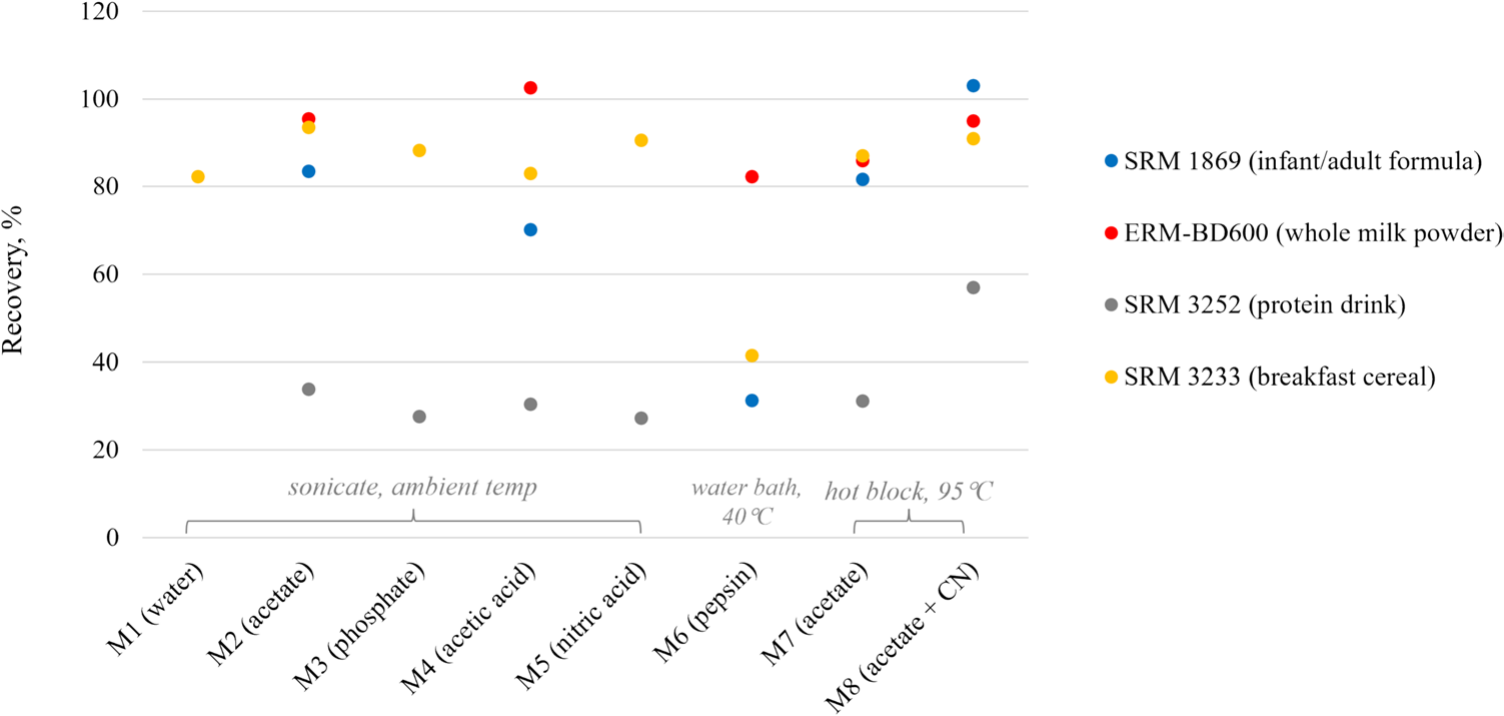
Vitamin B_12_ extraction from reference materials utilizing the extraction methods described in Table 3 (*n* = 2). Missing points correspond to samples that could not be analyzed by HPLC-ICP-MS due to unclarified extracts

Figure 2 summarizes the percentage of vitamin B_12_ extracted from the four reference materials using the methods in Table 3. Recoveries could not be determined for some of the aqueous, phosphate, and nitric acid extractions (missing points in Fig. 2) as they did not provide clarified extracts. None of the eight methods gave a result that agrees with the value reported on the certificate of analysis for SRM 3252 (protein drink mix) which will be explained under single laboratory validation of the method. The performance of all conditions, except enzymatic extraction (M6), was satisfactory for SRM 3233 (breakfast cereal). Enzymatic extraction did not yield notable benefits for any of the reference materials. Sodium acetate (M2, M7 and M8) extractions provided acceptable recoveries for all the materials except SRM 3252. A closer look at the performance of these three methods suggests that some forms of vitamin B_12_ may be lost when extractions are performed without the use of cyanide. Addition of cyanide resulted in enhanced extraction, leading to the selection of M8 as the optimal condition for routine analysis.

The effect of heating duration was assessed for the selected condition (M8) by extracting the four reference materials for periods of 30, 45, 60, 90, and 120 min. No significant differences were observed in extraction yield across the specified time frame, demonstrating that prolonged heating did not increase the risk of analyte degradation at 95 °C. The extraction time was maintained at 30 min to enhance sample throughput of the method. The conversion efficiency of cobalamins to CNCbl was further evaluated by treating standard solutions of OHCbl, MeCbl, and AdoCbl with sodium cyanide. The standards were prepared in sodium acetate buffer and processed following the optimum sample preparation procedure. Analysis of the samples using a cobalamin speciation analysis HPLC-ICP-MS method (unpublished) demonstrated that all the three cobalamin species were quantitatively transformed to CNCbl within the optimal quantity of reagent and extraction time.

### Analyte Enrichment and Cleanup

**Fig. 3:**
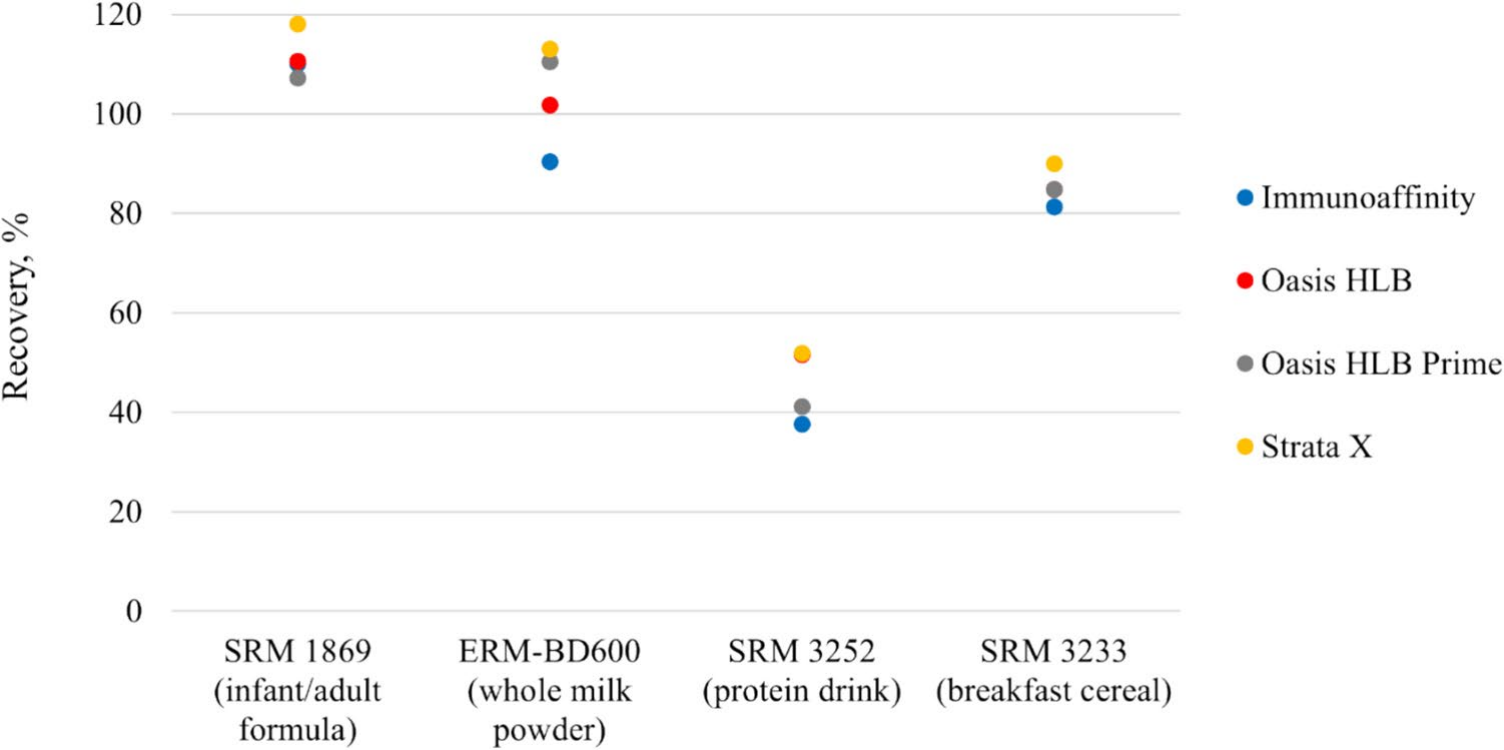
Vitamin B_12_ preconcentration and cleanup on four SPE materials from acetate extracts generated from reference materials (*n* = 2)

Low mass fractions of vitamin B_12_ in samples of the type considered in this study necessitate preconcentrating the analyte for improved detection. A cleanup step by SPE also helps remove interfering components from the extract. Four SPE materials were compared for effective applications as described in earlier. EASI-Extract immunoaffinity column has been widely used to preconcentrate vitamin B_12_ extracted from infant formulas (AOAC 2014; Heudi et al. 2006), milk and dairy products (Dubascoux et al. 2021; Huang et al. 2022; Pakin et al. 2005), as well as breakfast cereals (Heudi et al. 2006). Several studies also showed the application of polymeric reversed-phase materials such as Oasis HLB and Strata-X on extracts generated from infant formulas (Lee et al. 2015), milk and dairy products (Rampazzo et al. 2022; Repossi et al. 2017; Zironi et al. 2013), and other matrices not considered in the present study (Czerwonka et al. 2014; D’Ulivo et al. 2017; Szterk et al. 2012). Acetate extracts prepared from four reference materials were processed through the four SPE sorbents as outlined in the experimental part. Figure 3 illustrates that, except for a few outliers, the sorbents exhibited comparable performance across all reference materials.

**Fig. 4:**
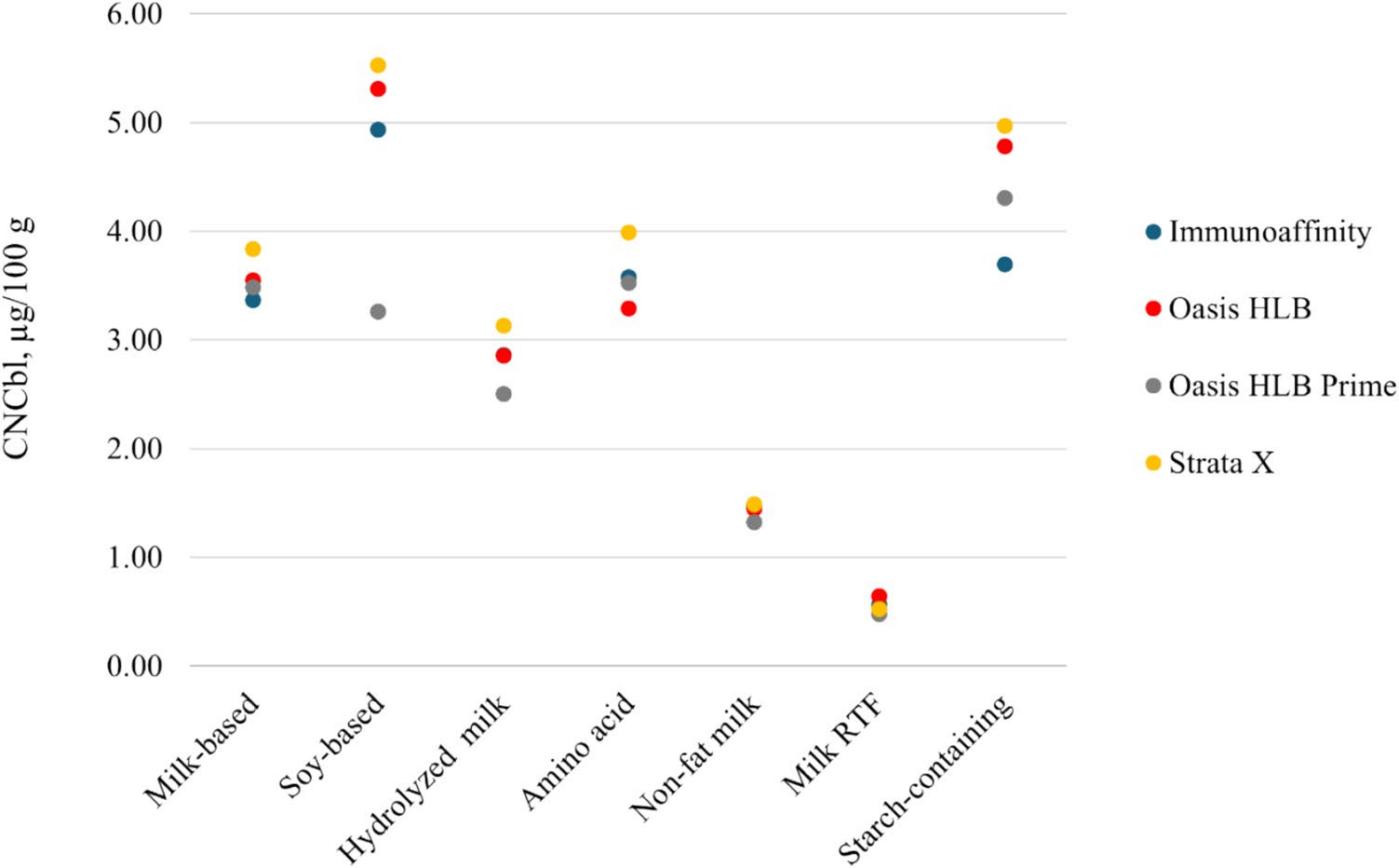
Vitamin B_12_ preconcentration and cleanup on four SPE materials from acetate extracts generated from representative infant formulas of different matrix types (*n* = 2)

To further explore the impact of matrix components, extracts generated from milk-based, soy-based, partially hydrolyzed milk-based, amino acid-based, starch-containing, and ready-to-feed infant formulas were processed. Apart from Oasis Prime HLB, which retained significantly lower fractions of CNCbl from the soy-based formula, the results were mostly consistent across the sorbents; see Fig. 4. Because of its efficient analyte retention, operational simplicity, and affordability, Oasis HLB column was selected for use.

### Stability of CNCbl

CNCbl is recognized for having a more stable chemical structure than the other forms of vitamin B_12_, but literature presents conflicting information about its stability in solutions (Bosle et al. 2016; Raju et al. 2013). The stability of CNCbl was examined in acetate extracts generated from reference materials. Extracts were prepared in amber tubes in duplicate following the optimum extraction procedure, with one set maintained at room temperature and the other set preserved at 4 °C. Mass fraction of CNCbl was measured in the extracts on the day of extraction (Day 0) and on Days 1, 2, 3, and 8 post-extraction. The percentage of CNCbl lost in the extracts in those days ranged between 0.1 and 6.3%, showing extracts can be preserved away from light for at least 8 days without the risk of losing a significant amount of CNCbl. Nevertheless, improper handling may lead to analyte loss.

### Single Laboratory Validation

The proposed method was single laboratory validated in accordance with FDA Guidelines for Validation of Chemical Methods (FDA 2019). Care was taken in the selection of samples to represent the disparate matrix categories across nutritional products (Table 1). Nine reference materials representing the product groups were included. Method performance characteristics evaluated during the validation study included LOD and LOQ, repeatability, accuracy (reference material and spike recovery), and measurement uncertainty. The interday precision of the method was evaluated by preparing and analyzing extracts over the course of three days.

#### Limit of Detection and Quantification

Analytical solution detection and quantitation limits (ASDL and ASQL, respectively) were determined based on the standard deviation of the replicate data (*n* = 10) for a fortified blank (FDA 2021). LOD and LOQ were calculated by multiplying the ASDL and ASQL by the preconcentration factor, respectively. Vitamin B_12_ can be detected and quantified using the current method at levels as low as 0.004 and 0.03 µg CNCbl/100 g (liquid and ready-to-feed/drink samples) and 0.01 and 0.06 µg CNCbl/100 g (solid samples), respectively, after preconcentrating the analyte on an Oasis HLB SPE sorbent.

#### Repeatability

Repeatability, expressed as RSD (%), was evaluated based on data from the interday preparation and analysis of extracts over three days (*n* = 3). Table 1 summarizes the average mass fractions of CNCbl along with the RSD (%) values for mass fractions above LOQ. The RSD values were below 14%, demonstrating good repeatability for analysis of replicate extracts prepared over several days.

#### Accuracy

The accuracy of the proposed method is determined based on the analysis of nine reference materials and 38 fortified samples.

- SRM Recovery

**Fig. 5:**
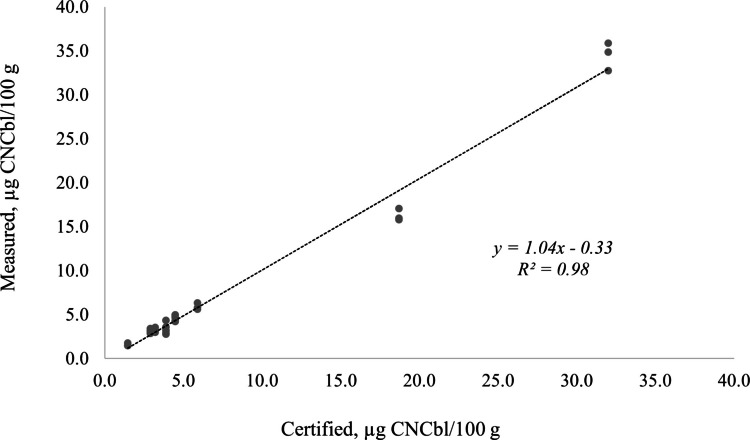
Correlation plot for measured CNCbl mass fraction (µg/100 g) in reference materials (excluding SRM 3252) against their certified values

The measured mass fractions of vitamin B_12_ for the reference materials ranged between 71 and 121% of the certified or reference values. Of all the replicate measurements for the reference materials (except SRM 3252), only two replicates of SRM 1846, which was analyzed past its expiration, were outside the range of 80–120%. Recovery, however, was significantly low (51%) for SRM 3252 (see discussion below). The correlation plot in Fig. 5 shows very good agreement between the measured and certified values (SRM 3252 not included). *Z*-scores, calculated according to FDA’s Elemental Analysis Manual Section 3 (FDA 2021), ranged from −3.6 to 1.7 with a median of −0.3. Other than two outliers (−3.6 and −3.1), corresponding to two SRM 1846 replicates, all the *Z*-scores were between −2 and 2 at 10% total uncertainty showing that the described method features an uncertainty no worse than 10% for the sample matrices evaluated.

The authors were unable to locate any literature data for vitamin B_12_ in SRM 3252 to compare with the result from the current study. Additional experiments were performed to identify possible reason(s) for the poor recovery of vitamin B_12_ from the SRM. To confirm complete conversion of the analyte to CNCbl during extraction, the extract was analyzed using a cobalamin speciation analysis HPLC-ICP-MS method (unpublished). The SRM was also fortified with CNCbl and extracted to evaluate the effect of the extraction condition on the recovery of the analyte from this matrix. No other cobalamin variant was detected in the extract, and the spiked CNCbl was recovered quantitatively. Finally, the SRM was sent to two external laboratories as a blind sample for analysis using microbiological techniques. The reported results were 103% and 115% of the result obtained in the current validation, bolstering the reliability of the validation results. The finding was communicated to NIST, and the reference value was retracted from the certificate of analysis of the SRM. Recertification of SRM 3252 or production of another protein drink reference material is called upon as it provides a useful and mandatory tool in method validation and analysis of samples.

b) Spike Recovery

**Fig. 6:**
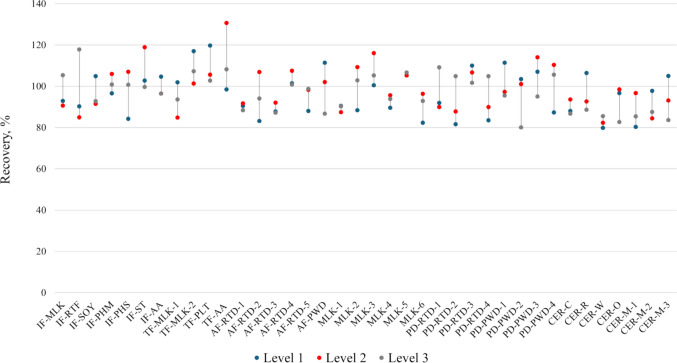
Recovery (%) of CNCbl fortified into the samples listed in Table 1. Mean values are presented for duplicate (*n* = 2) preparations of extracts at three spike levels. The recoveries for the three-tier spikes of a sample are connected by a vertical line

All the commercial products in Table 1 were used for spike recovery testing. Figure 6 summarizes mean recoveries of CNCbl for duplicate preparation of extracts at three spike levels. Fortification recoveries were within 100 ± 20%, except one outlier (131%). The results verified that the optimum extraction condition was effective for vitamin B_12_ recovery in different matrix types.

### Comparison with Other Methods

To corroborate the validation results, the proposed method was compared against AOAC Official Method 952.20 (microbiological assay) (AOAC 1960) through the analysis of the protein drink/powder and breakfast cereal samples in Table 1 by external laboratories. Reference materials were provided as blind samples for control purposes. The method was also compared with AOAC Official Method 2014.02 (UPLC-UV) (AOAC 2014) by analyzing the infant formula and toddler/adult nutritive drinks in Table 1. The HPLC-ICP-MS and UPLC-UV analyses were performed in the same laboratory by different analysts.

**Fig. 7:**
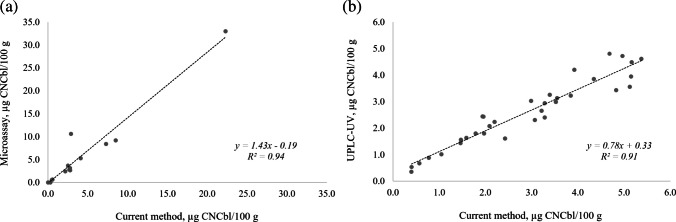
Correlation plots showing comparison of methods for CNCbl (µg/100 g) measurement in **a** protein drinks/powders and breakfast cereals and **b** infant formulas and toddler/adult nutritive drinks

Linear regressions between the measured CNCbl mass fractions in the two sets of comparisons are shown in Fig. 7. Both comparisons demonstrated good linearity with *R*^2^ > 0.90. The microbiological assay overestimated the vitamin B_12_ level with a slope of 1.4, while the UPLC-UV method showed slight underestimation (slope = 0.8). The microbiological assay is known to suffer from poor selectivity because the bacteria that grow in proportion to the amount of vitamin B_12_ (*Lactobacillus delbrueckii*) are also sensitive to species structurally similar to vitamin B_12_ leading to overestimated results (Li et al. 2019). For samples with very low analyte levels, small differences in measurement between methods result in proportionally high differences, explaining the slight underestimation of vitamin B_12_ by UPLC-UV.

The current method shows acceptable accuracy, specificity, and repeatability as defined by AOAC Standard Method Performance Requirements for Vitamin B_12_ in Infant Formula and Adult/Pediatric Nutritional Formula* (*SMPR 2011.005) (AOAC 2011). To our knowledge, the method covers a broader spectrum of matrices compared to methods currently available. Comparisons also demonstrated its advantages over most official and other published methods for nutritional products (Table 4). Its LOD and LOQ were lower than reported methods based on HPLC-UV (Choi et al. 2003; Heudi et al. 2006), HPLC-ICP-MS (Dubascoux et al. 2021; Raju et al. 2013; Yang et al. 2024), and HPLC with tandem mass spectrometry (HPLC-MS/MS) (Huang et al. 2022; Lee et al. 2015; Zironi et al. 2013). HPLC-MS/MS methods often have high sensitivity and specificity for most analytes including cobalamins (Li et al. 2019), yet ICP-MS demonstrates more robustness to handle complex matrixes. The current method also possessed the advantage of less sample requirement (Amritkar et al. 2020; AOAC 1960, 2014; Heudi et al. 2006; Huang et al. 2022; Oprean et al. 2011; Zironi et al. 2013), and shorter extraction (Amritkar et al. 2020; Heudi et al. 2006; Oprean et al. 2011) and analysis times (Amritkar et al. 2020; AOAC 1960; Choi et al. 2003; Heudi et al. 2006; Huang et al. 2022; Lee et al. 2015; Oprean et al. 2011; Raju et al. 2013) compared to most existing methods for samples of the type considered in the current study. Extraction was performed in sealed centrifuge tubes and avoids open and unsafe glassware used elsewhere (Amritkar et al. 2020; AOAC 2014; Heudi et al. 2006; Huang et al. 2022). A more affordable SPE material was used for analyte enrichment and cleanup.

**Table 4:** Comparison of analytical performances between the current and some existing methods

**Sample matrix**^**a**^	**Sample size**	**Extraction method, time**^**b**^	**SPE material**	**Analysis method, time**	**LOD, LOQ**	**Reference**
Infant formula, toddler/adult nutritive drinks, milk, protein drinks, breakfast cereals	0.5–5 g	Heating, 30–60 min	Oasis HLB C-18	HPLC-ICP-MS, 6 min	0.004, 0.03 (liquid) and 0.01, 0.06 µg/100 g (solid)	current method
Infant formula, adult/pediatric nutritional formula	25 g	Heating, 45 min	Immunoaffinity	UPLC-UV, 8 min HPLC-UV, 16 min	n/a	(AOAC 2014)
Milk	1 mL	Heating, 30 min	Immunoaffinity	HPLC-ICP-MS, 6 min	n/a, 54 ng/L	(Dubascoux et al. 2021)
Milk powder	2 g	Sonication, 40 min	n/a	HPLC-ICP-MS, 4 min	n/a, 2.11 µg/kg	(Yang et al. 2024)
Infant and pediatric formulas and adult nutritionals	3–10 g	Heating, 60–90 min	Supelco C-18	HPLC-UV, 33 min	n/a	(Amritkar et al. 2020)
Breakfast cereals	2 g	Shaking & sonication, 30 min	n/a	HPLC-ICP-MS, 30 min	0.9 ng/g, n/a	(Raju et al. 2013)
Fortified foods	Equivalent to 200 ng vitamin B_12_	30 min	n/a	HPLC-UV/vis, 32 min	2 µg/kg, n/a	(Choi et al. 2003)
Food products and premixes	5–25 g	3 h 35 min	Immunoaffinity	LC-UV, 22 min	3 ng/mL, n/a	(Heudi et al. 2006)
Infant and toddler milk formulas	1 g	15 min	Oasis HLB C-18	HPLC-MS/MS, 40 min	0.02 ug/g, n/a	(Lee et al. 2015)
Milk	20 g	Sonication, 70 min	n/a	LC-UV, 20 min	n/a	(Oprean et al. 2011)
Milk and dairy products	10 g	60 min	Oasis HLB C-18	HPLC-MS/MS, 2 min	n/a, 2 ng/g	(Zironi et al. 2013)
Milk and dairy products	30 g	60 min	Immunoaffinity	HPLC-MS/MS, 7 min	0.5, 1.0 µg/kg	(Huang et al. 2022)

^b^Only methods that were developed for nutritional products were considered for comparison

^a^Only the liquid extraction steps were compared, as the timing for SPE were not specified in the studies

The underlined numbers represent the experimental parameters that have been improved in the current method in comparison to the method under consideration

### Vitamin B_12_ in the Commercial Products

Table 1 compares the label claimed and measured mass fractions of vitamin B_12_ in the tested commercial products. The analytical results were higher than the values declared by the manufacturers by factors of 1.7–3.8 (infant formulas), 1.0–19.5 (toddler and adult nutritive drinks), 1.7–2.3 (nondairy milks), 1.3–5.4 (protein drinks/powders), and 1.0–10.5 (breakfast cereals). Exceptions were dairy and oat milks, for which the measured values were lower by factors of 0.5–0.7.

Excessive levels (referred to as overage amount) of vitamin B_12_ (Greibe and Nexo 2016) and other vitamins (Pehrsson et al. 2014) have been reported in infant formulas. Manufacturers may design their products to include nutrients in quantities that exceed the label claims to compensate for the inherent variability of the manufacturing process and limitations associated with analytical methods used for product testing. Overformulation also aims to counteract potential degradation of the vitamin over the product’s shelf life and to guarantee that the product retains its potency throughout that duration (Pharmacopeia 2016). Measured values for dairy milks lower than the label claims might be explained because those products are not fortified. Rather, vitamin B_12_ mass fractions in dairy products are naturally occurring and may be more susceptible to variation depending on the nutrient stability, type of product, and how it is processed.

The levels of vitamin B_12_ found in the tested infant formula samples comply with the minimum requirement of 0.15 µg/100 kcal as stipulated by the Code of Federal Regulations Title 21 (CFR 2015). At present, there is no defined upper limit for vitamin B_12_, as the body efficiently eliminates any surplus of this water-soluble nutrient, rendering it highly unlikely to attain a toxic level.

### Total Cobalt

The products in Table 1 (except milk) were analyzed for total their Co content. The results demonstrated that only a fraction of their total Co was in the form of vitamin B_12_, i.e., 3–16% (infant formulas), 1–68% (toddler and adult nutritive drinks), 3–79% (protein drinks/powders), and 4–28% (breakfast cereals). A report from the European Food Safety Authority (EFSA) Panel on Scientific Opinion indicated that certain food products may contain significant levels of Co that are not associated with vitamin B_12_, reaching as high as 70–95% in milk (EFSA 2009). Noncorrin Co-containing enzymes are known to exist in matrices such as proteins in minimal quantities. Additionally, Co in forms other than vitamin B_12_ can be sourced from matrix ingredients such as plants. The purity of CNCbl added to the products also plays a significant role.

## Conclusion

A method has been developed and validated for the determination of vitamin B_12_ in nutritional products offering an adequate analytical solution to measurement needs. The method is simpler and less time-consuming than most official and other published methods for nutritional products, with better analytical performance, broader application, and potential extension to other matrices. Collective extraction and enrichment of vitamin B_12_ as CNCbl were necessary for analyte stability and improved detection. The method demonstrated the merits of coupling reversed phase HPLC with ICP-MS for vitamin B_12_ detection, providing a simplified chromatogram compared to other common detection techniques and offering the advantages of high sensitivity and less interference. The short analysis time further increases sample throughput. The validation results of the current method demonstrated that the method is sensitive, selective, and robust to determine vitamin B_12_ in nutritional products, thus serving as a valuable tool for quality control. To the authors’ knowledge, the presented validation exercise is more rigorous than most previous reports in the same field, as it was exhaustive in covering a broad spectrum of matrices and levels of analyte. The validation also addressed the day-to-day variability of the method by preparing and analyzing extracts over several days. Comparison with microbiological assay and UPLC-UV further validated the method for its intended use. Cyanide is the sole reagent identified so far to efficiently convert cobalamins into a stable form. Since it presents safety hazards in practice, the outlined safety protocols should be strictly followed. The authors believe that future research will focus on identifying a safe alternative for converting labile cobalamins into a stable form.

## Data Availability

No datasets were generated or analysed during the current study.
